# A multicenter study of bodily distress syndrome in Chinese outpatient hospital care: prevalence and associations with psychosocial variables

**DOI:** 10.1186/s12888-022-04342-y

**Published:** 2022-11-24

**Authors:** Jing Ma, Linli Zheng, Ran Chen, Jie Ren, Hua Chen, Yaoyin Zhang, Wentian Li, Xiquan Ma, Wei Lu, Heng Wu, Kurt Fritzsche, Anne Christin Toussaint, Rainer Leonhart, Jing Wei, Lan Zhang

**Affiliations:** 1 Mental Health Center, West China Hospital, Sichuan University, Chengdu, China; 2 Mental Health Center, University-town Hospital, Chongqing Medical University, Chongqing, China; 3 Department of Rehabilitation, General Hospital of Jincheng Anthracite Coal Mining Group Co. Ltd, Jincheng, China; 4 Department of Psychological Medicine, Zhong Shan Hospital, Fudan University, Shanghai, China; 5 Department of Psychosomatic Medicine, Sichuan Provincial People’s Hospital, University of Electronic Science and Technology of China, Chengdu, China; 6 Department of Clinic Psychology, Wuhan Mental Health Centre, Wuhan, China; 7 Department of Psychosomatic Medicine, Dongfang Hospital, School of Medicine, Tongji University, Shanghai, China; 8 Department of Psychosomatic Medicine, Beijing Hospital of Traditional Chinese Medicine, Capital University, Beijing, China; 9 Department of Psychosomatic Medicine, Tongji Hospital, School of Medicine, Tongji University, Shanghai, China; 10 Department of Psychosomatic Medicine and Psychotherapy, Medical Center - University of Freiburg, Faculty of Medicine, Breisgau, Germany; 11 Department of Psychosomatic Medicine and Psychotherapy, University Medical Centre Hamburg-Eppendorf, Hamburg, Germany; 12 Institute of Psychology, University of Freiburg, Breisgau, Germany; 13 Department of Psychological Medicine, Peking Union Medical College Hospital, Chinese Academy of Medical Sciences and Peking Union Medical College, Beijing, China

**Keywords:** Bodily distress syndrome, Functional somatic symptoms, Psychosocial variables, Anxiety, Depression

## Abstract

**Background:**

Bodily distress syndrome (BDS) is a new, empirical-based diagnosis of functional somatic symptoms. This study aimed to explore the prevalence of BDS and its association with psychosocial variables in a Chinese clinical population.

**Methods:**

A multicentre cross-sectional study of 1269 patients was conducted in 9 different Chinese tertiary outpatient hospitals. The BDS was identified by trained interviewers face-to face, based on a brief version of the Schedules for Assessment in Neuropsychiatry (RIFD) and the BDS Checklist-25. Sociodemographic data and further information were characterised from psychometric questionnaires (The Patient Health Questionnaire-15, the Patient Health Questionnaire-9, the General Anxiety Disorder-7, the Whiteley scale-8) .

**Results:**

Complete data were available for 697 patients. The prevalence of BDS was 26.8% (95% confidence interval (CI): 23.5–30.1). Among the participants, 5.8% (95% CI: 4.1–7.6) fulfilled the criteria for single-organ BDS, while 20.9% (95%CI: 17.9–24.0) had multi-organ BDS. Comparison of the PHQ-15, PHQ-9, GAD-7, and WI-8 scores revealed higher scores on all dimensions for patients with BDS. In a binary logistic regression analysis, BDS was significantly associated with increased health-related anxiety (WI-8) and depression (PHQ-9). The explained variance was Nagelkerke’s *R*^2^ = 0.42.

**Conclusions:**

In China, the BDS is a common clinical condition in tertiary outpatient hospital settings with high prevalence, and is associated with health anxiety and depressive symptoms. In this clinical population, the severe multi-organ subtype of BDS was the most frequent.

## Background

Distressing physical symptoms that are difficult to explain in terms of well-defined physical diseases have traditionally been called ‘medically unexplained somatic symptoms’ [1]. These symptoms are found in approximately 33% of the cases in primary care settings and up to 50% of the cases in medical specialist consultations [2, 3]. Functional somatic symptoms are difficult to diagnose. Some doctors are likely to provide unnecessary diagnostic procedures to avoid missed diagnoses of medical disorders. However, excessive interventions may foster somatic fixation in patients, leading to the severe impairment of their social functioning, subjective suffering, and increased health care costs [4]. Specialised medicine used different diagnostic criteria to define and differentiate functional somatic symptoms. The most well-established diagnoses are fibromyalgia (for nonspecific muscular or skeletal pain), irritable bowel syndrome (for gastroenterological symptoms), and chronic fatigue syndrome (for chronic states of exhaustion) [5]. In the fifth edition of the Diagnostic and Statistical Manual of Mental Disorders (DSM-5) and in the Eleventh Revision of the International Classification of Disorders (ICD-11), somatoform disorders were replaced by somatic symptoms and related disorders (SSD) and bodily distress disorders (BDD), respectively. Meanwhile, the distinction between symptoms with and without underlying pathophysiology has been omitted [6–8]. Recently, Fink et al. proposed a new diagnostic category called the bodily distress syndrome (BDS) [9], the concept of the BDS was originally developed on the basic of empirical research conducted with the idea of establishing a unifying diagnostic category that could encompass the majority of functional disorders and syndromes. The hallmark of the BDS is that patients suffer from various physical symptoms of bodily distress. Therefore, this diagnostic category is defined not simply by listed symptoms but by specific symptom patterns.

The BDS has been shown to capture the most of functional somatic syndromes, including fibromyalgia, chronic fatigue syndrome, hyperventilation syndrome, irritable bowel syndrome, noncardiac chest pain, other pain syndromes, or any somatoform disorder [10, 11]. At least 90% of patients suffering from one of these disorders fulfil the criteria for BDS [11, 12].

As is divided into moderate, single-organ type (with four subtypes) and severe, multi-organ type, the BDS unite four symptom groups (gastrointestinal, cardiopulmonary, musculoskeletal, and general symptoms) and typically emerges in patterns of physical symptoms [13].While there was a lack of structured diagnostic interviews for the BDS in the past, based on previous large-scale empirical research, Fink proposed a revised version of the Schedules for Neuropsychiatric Assessment (SCAN) as a clinical diagnostic interview to assess BDS [9], which called research interview for functional somatic disorders and health anxiety (RIFD) [14]. However, to date, only few studies used this tool to investigate the prevalence of BDS, with samples limited to clinical populations in Denmark and Germany [15, 16]. Bringing BDS, this unite of symptom groups, into the clinical settings is not only providing a better description and explanation for patients, but also help preventing patients suffer related mental burdens by early detection,

In order to provide more clinical information on the BDS in China, we conducted a multicentre cross-sectional survey to investigate the prevalence of the BDS and its associations with psychosocial variables in Chinese patients from outpatient hospitals.

## Methods

### Design and procedures

A multicentre cross-sectional study was performed between September 2016 and January 2018 in nine outpatient clinics of general hospitals in Beijing, Shanghai, Chengdu, Wuhan, and Jincheng, located in northern, south-eastern, and south-western region of China. The neurology and gastroenterology departments of these hospitals were chosen to represent modern biomedical settings. The traditional medical settings collected Traditional Chinese medicine (TCM) departments. The departments of psychological medicine (PSY) were selected to represent psychosomatic and psychiatric centres.

All the hospitals invited were regarded as “3A hospitals”, indicating that they meet the highest standards in China. As comprehensive or general hospitals at the city, provincial, or national level, these hospitals are responsible for providing specialist health services, performing a more significant role with regard to medical education and scientific research, and serving as medical hubs that provide care to multiple regions.

Patients recruited in this study using convenience sampling. On randomly assigned days, all outpatients in these departments were consecutively informed about the study and invited to participate by research executives at various centres. Based on the power analysis, we aimed to recruit 220 patients from each of the above-mentioned medical settings.

### Study participants

The participants we included were aged 18+ years. All of them received written or oral information about the study and provided written informed consent. Patients who were diagnosed with schizophrenia, bipolar disorder, and severe mental disorders such as acute psychosis and suicidal tendencies, who had language difficulties, or who were unable to complete the interview and the questionnaires due to significant neurocognitive dysfunction were excluded.

### Measurements

#### Demographics

Data on demographical status were obtained by self-made questionnaire, including age, gender, marital state, ethnicity, living site, life status, education, household income, employment status, insurance, alcohol and smoking history and department.

#### Diagnostic assessment

The symptoms were screening by the Bodily Distress Syndrome Checklist-25 (BDS-25 checklist) while the RIFD were used to distinguish the subtypes [14]. Evaluating four physical symptom clusters including cardiopulmonary, gastrointestinal, musculoskeletal, and general symptoms, the RIFD had experienced the completed translate procedure. First, it was translated into Chinese by a language expert and was then reviewed and revised by two clinicians who had experience in mental disorders treatment, and later another expert back-translated the Chinese version into English to ensure its linguistic accuracy. The RIFD was conducted face to face through research assistants (students of psychology at the master’s level, students of medicine in their final year of study, and medical doctors) trained by experienced psychiatrists.

The BDS-25 checklist was used to assess the physical symptoms. This scale has 25 items, including four symptom clusters (cardiopulmonary, gastrointestinal, musculoskeletal, and general symptoms) [17–19].

Based on physical symptom groups, the BDS symptoms cannot be explained by an underlying physical disease. The BDS includes multi-organ subtype and single-organ subtypes. Single-organ BDS involves one or two symptom groups while multi-organ BDS involves three or four symptom groups [9]. The diagnostic criteria for BDS are presented in Table 1.

**Table 1:** Diagnostic criteria for BDS

Diagnostic criteria for BDS	
1) ≥ 3 symptoms from at least one of the following groups:	
• Cardiopulmonary/autonomic arousal: Palpitations /heart pounding, precordial discomfort, breathlessness without exertion, hyperventilation, hot or cold sweats, dry mouth	
• Gastrointestinal arousal: Abdominal pains, frequent loose bowel movements, feeling bloated/full of gas/distended, regurgitations, diarrhea, nausea, burning sensation in chest or epigastrium	
• Musculoskeletal tension: Pains in arms or legs, muscular aches or pains, pains in the joints, feelings of paresis or localized weakness, back ache, pain moving from one place to another, unpleasant numbness or tingling sensations	
• General symptoms: Concentration difficulties, impairment of memory, excessive fatigue, headache, dizziness.	
2) The patient has been disabled by the symptoms (i.e. daily living is affected)	
3) Relevant differential diagnoses have been ruled out	

#### Other psychological measurements

1. The Patient Health Questionnaire-15 (PHQ-15) was used to assess the presence and severity of common somatic symptoms within the last 4 weeks using 15 items, with the total scores range from 0 to 30 for women and from 0 to 28 for men. The Chinese version showed satisfactory reliability and validity [20].
2. The Patient Health Questionnaire-9 (PHQ-9) is a self-report instrument that indicates the depressive symptoms within the last 2 weeks. The total score ranges from 0 to 27. Previous studies have demonstrated good reliability and validity of this scale in Chinese general hospital outpatients [21, 22].
3. The General Anxiety Disorder-7 (GAD-7) was used to evaluate the severity of anxiety symptoms. Total scores ranging from 0 to 21. The reliability and validity of the GAD-7 has been verified in Chinese version [23].
4. The Whiteley scale-8 (WI-8) is a brief self-administrated tools that demonstrates the distress of patients with health-related anxiety symptoms over the past 4 weeks. It has eight items and each item is rated on a five-point Likert scale ranging from 1 to 5. The original well-validated seven-item scale (WI-7) was extended by one additional item: “Recurring thoughts about having a disease that are difficult to be rid of?” The WI-8 was first used in a Dan ish study of functional disorders [24]. High scores reflect high anxiety about health [25]. This instrument has shown confirmed reliability and validity in past years [26].

The other psychological measurements were all accessed self-reported.

### Statistical analysis

Data from our study were analysed using SPSS 26.0. To examine how many patients met a BDS diagnosis in the three different departments, the prevalence and 95% CI were calculated. We used the chi-square test to analyse the prevalence in the three groups. If there was a difference, the Bonferroni method was used to compare the incidence in pairs. To examine the characteristics related to the prevalence of BDS, we used the chi-square test to analyse categorical data. We used two-sample t-tests to analyse the scaled data. A backward stepwise binary logistic regression analysis was performed to explore the potentially influencing factors of BDS. We chose our predictors of interest based on previous studies from the field of research on somatoform disorders and somatic symptom disorders. Based on theoretical considerations derived from literature, we chose somatic symptom severity, symptom duration, depression, anxiety, health anxiety, doctor visits, and impairment in daily life as potential statistical predictors of BDS [27, 28].

## Results

### Patient characteristics

In total, 1269 patients were invited to participate the study. Among them, 699 participants (55.08%) completed questionnaires and only the data of the 697 patients who completed both the questionnaires and structured clinical interview were sent to the final analysis. A total of 572 patients were excluded for various reasons. Two patients failed to complete the interview, 266 patients refused to participate for the lack of time, 148 reported interest no interest, 34 had bad health status like fracture and cerebral infarction, 54 refused to participate for other reasons. Thirteen patients were excluded for visiting for others, 8 patients were excluded due to communication difficulties, 35 were only picking up prescriptions for relatives, 9 patients were disqualified for cognitive impairment, and 3 for acute suicidal tendency and severe psychosis.

### Prevalence of BDS

**Fig. 1:**
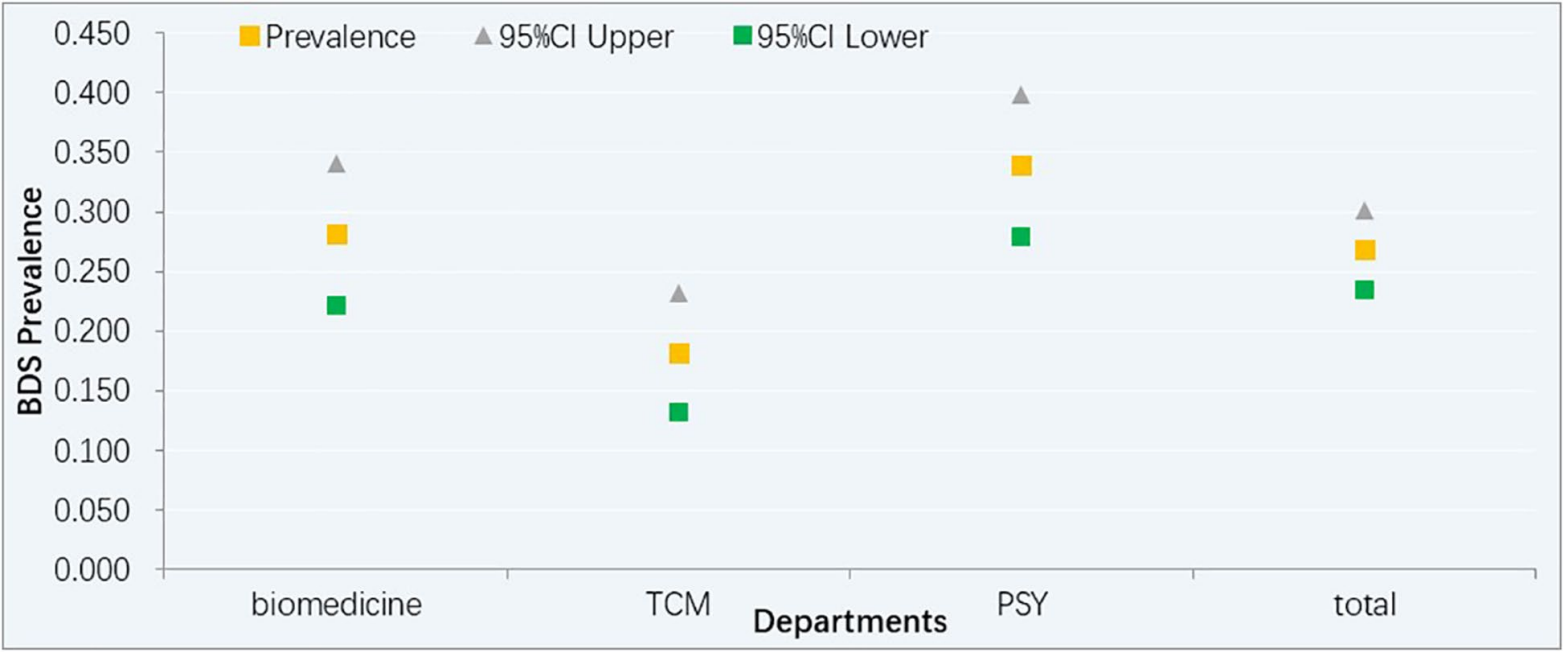
BDS prevalence in different departments. Note: Bodily Distress Syndrome (BDS), Traditional Chinese medicine departments (TCM), Psychosomatic medical settings (PSY)

Among the 697 patients, 187 patients fulfilled the criteria for BDS, rounding out its total prevalence at 26.8% (95%CI:23.5–30.1). The prevalence of BDS in the biomedicine, TCM, and PSY departments was 28% (95% CI: 22.2–34.1%), 18% (95% CI:13.2–23.2), and 33% (95%CI: 27.9–39.9), respectively. And there was a statistically significant difference in the prevalence of BDS among the three departments (Fig. 1). The single-organ BDS prevalence was 5.8% (95% CI 4.1–7.6%) while the multi-organ BDS prevalence was 20.9% (95% CI 17.9–24.0%).

### Comparison of characteristics between BDS patients and no BDS patients

There were no significant differences in ethnicity, living site, insurance, marital status, life status, education, income, employment status, alcohol consumption, and smoking status. The average age of patients with BDS was 42 (SD = 14.61), and 64% (119/187) of the patients with BDS were female (Table 2).

**Table 2:** Characteristics of included patients

	All	BDS-(510)	BDS+(187)	*χ*^*2*^*/t*	*p*
**Patients**					
**Age**	42.94 ± 14.23	43.21 ± 14.10	42.19 ± 14.61	0.839	0.573
**Gender**					
Male	38.59%	39.41%	36.36%	0.536	0.464
Female	61.41%	60.59%	63.64%		
**Marital state**				2.373	0.126
Single	27.26%	25.69%	31.56%		
Married	72.74%	74.31%	68.44%		
**Ethnicity**				0.911	0.340
Han	92.97%	93.53%	91.44%		
Others	7.03%	6.47%	8.56%		
**Living site**				1.327	0.249
City	82.21%	83.33%	79.57%		
Country	17.79%	16.67%	20.43%		
**Life status**				1.285	0.257
Living alone	9.18%	8.43%	11.23%		
Living with others	90.82%	91.57%	88.77%		
**Education**				4.439	0.218
Primary school	6.45%	5.29%	9.63%		
Middle school	19.66%	19.61%	19.79%		
High school	25.97%	26.67%	24.06%		
University and above	47.91%	48.43%	46.52%		
**Household income (RMB)**				4.680	0.096
< 4000	33.82%	31.49%	40.11%		
4000–8000	34.97%	35.84%	32.62%		
≥8000	31.21%	32.67%	27.27%		
**Employment status**				3.860	0.049
Employed	48.92%	51.17%	42.78%		
Unemployed	51.08%	48.82%	57.21%		
**Insurance**				2.034	0.154
Yes	86.94%	88.07%	83.96%		
No	13.06%	11.93%	16.04%		
**Alcohol**				0.233	0.972
Never	49.71%	49.31%	50.80%		
Social drinking	42.10%	42.63%	40.64%		
Drink in the past, but quit now	5.60%	5.50%	5.88%		
Almost drink everyday	2.59%	2.56%	2.68%		
**Smoking**				0.271	0.873
Never	72.56%	72.89%	71.66%		
In the past	13.07%	13.16%	12.83%		
Currently	14.37%	13.95%	15.51%		
**Department**				15.126	0.001
TCM	33.14%	37.06%	22.46%		
Biomedicine	32.14%	31.57%	33.69%		
PSY	34.72%	31.37%	43.85%		

Bodily Distress Syndrome (BDS), Traditional Chinese medicine departments (TCM), Psychosomatic medical settings (PSY)

### Assessment score of BDS

Comparing the PHQ-15, PHQ-9, GAD-7, and WI-8 scores between patients with and without BDS, patients with BDS reported a higher score on all dimensions (Table 3). In the stepwise regression (backward), the GAD-7 questionnaire was removed from the regression equation because its regression weight was not significant. The odds of PHQ-9, WI-8, and PHQ-15 were as follows: 1.041, 95% CI 1.007–1.076, *P* = 0.018; 1.098, 95% CI 1.067to 1.130, *P* < 0.0001; 1.184, 95% CI 1.129 to 1.242, *P* < 0.001, respectively. The explained variance was Nagelkerke R-square = 0.42 (Table 4). Thus, the BDS was significantly associated with increased health-related anxiety (WI-8), depression (PHQ-9), and somatic symptoms (PHQ-15).

**Table 3:** BDS and somatic symptom severity, depression, general anxiety and health anxiety

Total score	BDS(−)		BDS(+)		*t*	*p*
	n	Mean (SD)	n	Mean (SD)		
PHQ-15	510	7.74 ± 4.41	187	13.73 ± 5.36	−13.591	< 0.0001
PHQ-9	510	7.53 ± 6.06	187	11.85 ± 7.16	−7.337	< 0.0001
GAD-7	510	5.66 ± 5.36	187	9.37 ± 6.43	−7.046	< 0.0001
WI-8	510	15.77 ± 6.72	187	25.03 ± 8.69	−13.188	< 0.0001

number(n), The Patient Health Questionnaire-15 (PHQ15), The Patient Health Questionnaire-9 (PHQ-9), The General Anxiety Disorder-7 (GAD-7), The Whiteley scale −8 (WI-8)

**Table 4:** Results of multiple logistic backward regression analysis to predict BDS diagnosis

		B	Std. Error	Wald	df	Sig.	Exp (B)	95%Cofidengce Interval of EXP(B)	
								Lower Bound	Upper Bound
Step 1	PHQ9	0.028	0.025	1.287	1	0.257	1.029	0.980	1.080
	GAD7	0.017	0.028	0.393	1	0.531	1.017	0.964	1.074
	WI8	0.093	0.015	40.467	1	< 0.0001	1.097	1.066	1.129
	PHQ15	0.169	0.024	48.362	1	< 0.0001	1.185	1.129	1.243
	constant	5.061	0.372	185.483	1	< 0.0001	0.006		
Step 2	PHQ9	0.040	0.017	5.564	1	0.018	1.041	1.007	1.076
	WI8	0.094	0.014	42.034	1	< 0.0001	1.098	1.067	1.130
	PHQ15	0.169	0.024	48.493	1	< 0.0001	1.184	1.129	1.242
	constant	5.065	0.372	185.821	1	< 0.0001	0.006		

The Patient Health Questionnaire-15 (PHQ15), The Patient Health Questionnaire-9 (PHQ-9), The General Anxiety Disorder-7 (GAD-7), The Whiteley scale −8 (WI-8)

## Discussion

To our knowledge, this is the first multicentre study with a large sample size conducted to investigate the prevalence and characteristics of the BDS in Chinese population. The present study revealed an overall BDS prevalence rate of 26.8% across all centres, while the prior studies have shown that the prevalence rate of the BDS ranges from 12 to 36%, with the single-organ subtype being more frequent [9, 28, 29]. Notably, the results in our study revealed an opposite situation, that the multi-organ subtype of the BDS reported more frequent than the single-organ subtype. Several reasons may contribute to this difference. First, the samples collected in this study come from third-grade hospitals in China, where gathered patients who have a higher incidence of physical diseases than the general population and thus may have difficulty in getting diagnosed and treated in primary centres. The sample cluster may lead to a higher incidence. Second, previous studies have found that functional somatic disorders may be associated with cultural beliefs and social health education [30]. Compared to the culture and customs in Europe, Chinese people are generally not good at expressing emotions and are less likely seeking for help for mental issues. Instead, they tend to express their feelings indirectly by describing physical symptoms, which may account for the higher ratio of multi-organ subtypes [31]. These culture differences might remind us developing related treatments more suited the patients’ needs [6].

Beutel et al. have reported that the majority age of BDS patients ranged from 41 to 65 years [32], which was consistent with our results (the BDS patients age was 42 ± 14.61). Additionally, PSY department reported higher BDS prevalence that in the TCM department. This might be explained by the fact that the patients with the BDS usually have unexplained somatic symptoms and are referred to the psychiatric departments by doctors from various departments. The significantly higher BDS-25 checklist total score in the PSY department could demonstrated the situation. What’s more, most patients who visited the TCM department reported mild symptoms, that may due to the nature of the department for TCM department is much more like a primary health centre that treat the normal physical distress.

There seems to be no significant difference in the gender composition between the groups. Same results have been found in the Danish study [31]. Nevertheless, recent studies have reported controversial results about the association between somatic symptom burden and sociodemographic factors. Beutel et al. has verified the association, including higher age, lower education, social and economic status, unemployment, and disruption of marriage relationship [32]. In the contrast, Cao et al. [33] showed that there were no differences in sociodemographic and lifestyle data between SSD and non-SSD patients. In accordance with the present study, no risk factors were found for ethnicity, living site, insurance, marital status, education, income, alcohol, employment status, or smoking status. The high rate of urban occupancy rate may result in this finding, for the low heterogeneity of the sample. What’s more, the sample source of this study collected from large cities with higher Gross Domestic Product, higher quality of population and higher insurance coverage than national average. Thus, the expected differences in sociodemographic are not shown.

The multiple regression results shown that for every point increase in the WI-8, PHQ-9, and PHQ-15 scores, the risk of being diagnosed with BDS increases, which is consistent with previous studies [11, 14]. The results suggested that BDS patients suffered higher risk of depression and healthy anxiety compared to the control group, which is not surprised because depression and BDD are comorbid frequently, and there is a substantial overlap between depression and somatisation [14]. Furthermore, depression and somatisation may emerge from shared psychosocial and biological diatheses [14, 33]. Some studies have suggested that many of the phenomena of somatoform disorders are associated with low threshold clustering of psychiatric syndromes or their atypical manifestations [34].

### Limitations

This study has several limitations. Because of the cross-sectional nature of our study, causality could not be inferred. It should be noted that the research approach used a Western biopsychosocial model of illness. Therefore, the possible culture-specific characteristics may not have been identified.

Another limitation of this study is that all included participants were restricted to three outpatient departments in China, which might result in a low heterogeneity.

## Conclusions

In China, the BDS is a common clinical condition in tertiary outpatient hospital settings with high prevalence. In this clinical population, the severe multi-organ subtype of the BDS reported the most frequent and the BDS is associated with health anxiety and depressive symptoms. Our study provides a powerful support about paying attention to BDS in all departments of general hospitals as well as the outpost community. Trained clinicians supposed to increase the awareness of catching these symptoms and provide better description for their patients. Further steps might be focus on a clinical longitudinal study that includes more departments.

## Data Availability

The datasets generated during and/or analysed during the current study are available from the corresponding author on reasonable request. We are sorry that we can’t share the current data right now for some of the data were staying unpublished. We assure the authenticity and validity of the data in this research, without undue reservation.

## Abbreviations

BDS: Bodily distress syndrome
SSD: Somatic symptoms and related disorders
BDD: Bodily distress disorders
RIFD: Research interview for functional somatic disorders and health anxiety
TCM: Traditional Chinese medicine
PSY: Psychological medicine
PHQ-15: Patient Health Questionnaire-15
PHQ-9: Patient Health Questionnaire-9
GAD-7: General Anxiety Disorder-7
WI-8: Whiteley scale-8

## References

[1] Peveler R, Kilkenny L, Kinmonth AL (1997). Medically unexplained physical symptoms in primary care: a comparison of self-report screening questionnaires and clinical opinion. J Psychosom Res.

[2] van der Feltz-Cornelis CM, Elfeddali I, Werneke U, Malt UF, Van den Bergh O, Schaefert R, Kop WJ, Lobo A, Sharpe M, Sollner W (2018). A European research agenda for somatic symptom disorders, bodily distress disorders, and functional disorders: results of an estimate-talk-estimate Delphi expert study. Front Psychiatry.

[3] Burton C, Fink P, Henningsen P, Löwe B, Rief W (2020). Functional somatic disorders: discussion paper for a new common classification for research and clinical use. BMC Med.

[4] Bener A, Ghuloum S, Burgut FT (2010). Gender differences in prevalence of somatoform disorders in patients visiting primary care centers. J Prim Care Community Health.

[5] Hüsing P, Löwe B, Toussaint A (2018). Comparing the diagnostic concepts of ICD-10 somatoform disorders and DSM-5 somatic symptom disorders in patients from a psychosomatic outpatient clinic. J Psychosom Res.

[6] Rief W, Burton C, Frostholm L, Henningsen P, Kleinstauber M, Kop WJ, Lowe B, Martin A, Malt U, Rosmalen J (2017). Core outcome domains for clinical trials on somatic symptom disorder, bodily distress disorder, and functional somatic syndromes: European network on somatic symptom disorders recommendations. Psychosom Med.

[7] Gaebel W, Zielasek J, Reed GM (2017). Mental and behavioural disorders in the ICD-11: concepts, methodologies, and current status. Psychiatr Pol.

[8] Lam TP, Goldberg DP, Dowell AC, Fortes S, Mbatia JK, Minhas FA, Klinkman MS (2013). Proposed new diagnoses of anxious depression and bodily stress syndrome in ICD-11-PHC: an international focus group study. Fam Pract.

[9] Budtz-Lilly A, Schroder A, Rask MT, Fink P, Vestergaard M, Rosendal M (2015). Bodily distress syndrome: a new diagnosis for functional disorders in primary care?. BMC Fam Pract.

[10] Petersen MW, Schroder A, Jorgensen T, Ornbol E, Meinertz Dantoft T, Eliasen M, Benros ME, Fink P (2020). Irritable bowel, chronic widespread pain, chronic fatigue and related syndromes are prevalent and highly overlapping in the general population: DanFunD. Sci Rep.

[11] Petersen MW, Schröder A, Jørgensen T, Ørnbøl E, Dantoft TM, Eliasen M, Thuesen BH, Fink P (2020). The unifying diagnostic construct of bodily distress syndrome (BDS) was confirmed in the general population. J Psychosom Res.

[12] Fink P (2017). Syndromes of bodily distress or functional somatic syndromes - where are we heading. Lecture on the occasion of receiving the Alison creed award 2017. J Psychosom Res.

[13] Fink P, Schroder A (2010). One single diagnosis, bodily distress syndrome, succeeded to capture 10 diagnostic categories of functional somatic syndromes and somatoform disorders. J Psychosom Res.

[14] Petersen MW, Schröder A, Jørgensen T, Ørnbøl E, Dantoft TM, Eliasen M, Fink P (2019). RIFD - a brief clinical research interview for functional somatic disorders and health anxiety. J Psychosom Res.

[15] Petersen MW, Schröder A, Jørgensen T, Ørnbøl E, Dantoft TM, Eliasen M, Carstensen TW, Falgaard Eplov L, Fink P (2020). Prevalence of functional somatic syndromes and bodily distress syndrome in the Danish population: the DanFunD study. Scand J Public Health.

[16] Häuser W, Hausteiner-Wiehle C, Henningsen P, Brähler E, Schmalbach B, Wolfe F (2020). Prevalence and overlap of somatic symptom disorder, bodily distress syndrome and fibromyalgia syndrome in the German general population: a cross sectional study. J Psychosom Res.

[17] Budtz-Lilly A, Fink P, Ørnbøl E, Vestergaard M, Moth G, Christensen KS, Rosendal M (2015). A new questionnaire to identify bodily distress in primary care: the 'BDS checklist'. J Psychosom Res.

[18] Schmalbach B, Roenneberg C, Hausteiner-Wiehle C, Henningsen P, Brähler E, Zenger M, Häuser W (2020). Validation of the German version of the bodily distress syndrome 25 checklist in a representative German population sample. J Psychosom Res.

[19] Petersen MW, Rosendal M, Ørnbøl E, Fink P, Jørgensen T, Dantoft TM, Schröder A (2020). The BDS checklist as measure of illness severity: a cross-sectional cohort study in the Danish general population, primary care and specialised setting. BMJ Open.

[20] Lee S, Ma YL, Tsang A (2011). Psychometric properties of the Chinese 15-item patient health questionnaire in the general population of Hong Kong. J Psychosom Res.

[21] Xiong N, Fritzsche K, Wei J, Hong X, Leonhart R, Zhao X, Zhang L, Zhu L, Tian G, Nolte S (2015). Validation of patient health questionnaire (PHQ) for major depression in Chinese outpatients with multiple somatic symptoms: a multicenter cross-sectional study. J Affect Disord.

[22] Chen R, Wang Y, Yu J, Zhang L (2017). Evaluation of the reliability and validity of PHQ-9 in general hospital inpatients. Sichuan Mental Health.

[23] He X, Li C, Jie Q, Cui H (2010). Reliability and validity of a generalized anxiety disorder scale in general hospital outpatients. Shanghai Arch Psychiatry.

[24] Carstensen TBW, Ørnbøl E, Fink P, Pedersen MM, Jørgensen T, Dantoft TM, Benros ME, Frostholm L (2020). Detection of illness worry in the general population: a specific item on illness rumination improves the Whiteley index. J Psychosom Res.

[25] Tu CY, Liao SC, Liu CY, Chen TT, Chen IM, Lin KF, Huang WL (2016). Application of the Chinese version of the Whiteley Index-7 for detecting DSM-5 somatic symptom and related disorders. Psychosomatics.

[26] Chen Y, Fink P, Wei J, Toussaint AK, Zhang L, Zhang Y, Chen H, Ma X, Li W, Ren J, Lu W, Leonhart R, Fritzsche K, Wu H (2021). Psychometric evaluation of the Whiteley Index-8 in Chinese outpatients in general hospitals. Front Psychol.

[27] Rosendal M, Olde Hartman TC, Aamland A, van der Horst H, Lucassen P, Budtz-Lilly A, Burton C (2017). "medically unexplained" symptoms and symptom disorders in primary care: prognosis-based recognition and classification. BMC Fam Pract.

[28] Rosendal M, Bro F, Fink P, Christensen KS, Olesen F (2003). Diagnosis of somatisation: effect of an educational intervention in a cluster randomised controlled trial. Brit J General Pract.

[29] Toft T, Fink P, Oernboel E, Christensen K, Frostholm L, Olesen F (2005). Mental disorders in primary care: prevalence and co-morbidity among disorders. Results from the functional illness in primary care (FIP) study. Psychol Med.

[30] Löwe B, Gerloff C (2018). Functional somatic symptoms across cultures: perceptual and health care issues. Psychosom Med.

[31] Budtz-Lilly A, Vestergaard M, Fink P, Carlsen AH, Rosendal M (2015). Patient characteristics and frequency of bodily distress syndrome in primary care: a cross-sectional study. Brit J General Pract.

[32] Beutel ME, Wiltink J, Ghaemi Kerahrodi J, Tibubos AN, Brahler E, Schulz A, Wild P, Munzel T, Lackner K, Konig J et al. Somatic symptom load in men and women from middle to high age in the Gutenberg Health Study - association with psychosocial and somatic factors. Sci Rep. 2019;9(1):4610. 10.1038/s41598-019-40709-0 PMC6418216 30872625

[33] Cao J, Wei J, Fritzsche K, Toussaint AC, Li T, Jiang Y, Zhang L, Zhang Y, Chen H, Wu H (2020). Prevalence of DSM-5 somatic symptom disorder in Chinese outpatients from general hospital care. General Hospital Psychiatry.

[34] Kellner R (1994). Psychosomatic syndromes, somatization and somatoform disorders. Psychother Psychosom.

